# Electrochemical Methodology for Evaluating Skin Oxidative Stress Status (SOSS)

**DOI:** 10.3390/diseases7020040

**Published:** 2019-05-27

**Authors:** Pincemail Joël, Kaci Mouna-Messaouda, Cheramy-Bien Jean-Paul, Defraigne Jean-Olivier, Meziane Smail

**Affiliations:** 1 Department of Cardiovascular Surgery/CREDEC, Antioxidant Nutrition and Health Platform, University of Liège and CHU, Sart Tilman, 4000 Liège, Belgium; jp.cheramy@chuliege.be (C.-B.J.-P.); jo.defraigne@chuliege.be (D.J.-O.); 2 Institut Européen des Antioxydants, Oxystress Technologies, 1 rue Victor de Lespinats, 54230 Neuves-Maisons, France; mkaci@ie-antioxydants.com (K.M.-M.); smeziane@ie-antioxydants.com (M.S.)

**Keywords:** oxidative stress, skin, electrochemical detection, PAOT-Skin Score^®^

## Abstract

For the purpose of human disease prevention, several methods have been developed, and are still developing, to assess the oxidative stress status (OSS) of individuals. In the present paper, we describe an approach based on electrochemical detection able to evaluate skin oxidative stress status (SOSS) as a PAOT (Pouvoir AntiOxydant Total)-Skin Score^®^. Normal reference values for the PAOT-Skin Score^®^ were: 0–62.94 (*n* = 263). Intra- and inter-assay coefficients of variation were, respectively, 12.47 ± 4.29% and 7.0 ± 2.5%. Our technology showed increased skin antioxidant activity following topical application of reduced coeznyme Q_10_ cream or vitamin C intake as orange juice or supplements. Moreover, we found significant correlations between some blood oxidative stress biomarkers and the PAOT-Skin Score^®^ (γ-tocopherol/α-tocopherol ratio (r = 0.43, *p* = 0.020); copper (r = −0.42, *p* = 0.022); copper/zinc ratio (r = −0.49, *p* = 0.006), and lipid peroxides (r = −0.43, *p* = 0.002)). In addition to being non–invasive, the present electrochemical methodology is also not expensive, fast, and easy to use.

## 1. Introduction

According to Jones [1], oxidative stress (OS) has been defined as an imbalance between reactive oxygen species, or ROS (including free radical and non-free radical species), and antioxidants in favor of the former, leading to a disruption of the redox signaling and/or molecular damage to lipids, proteins, and DNA. Such a concept takes into account that ROS comprise both physiological and pathological properties [2,3]. At the physiological level, ROS act as secondary messengers (redox signaling) able to induce over-expression of gene coding for antioxidant enzymes. When produced in excessive amount, ROS, as powerful oxidants, provoke irreversible damage to lipids, DNA, and proteins, which are potentially involved in the development of ageing and ageing-associated diseases (cardio-vascular and neuro-degenerative pathologies, cancer, diabetes, etc.). Increased OS may result from a poor diet in fruits and vegetables known for their richness in antioxidants but also from endogenous and exogenous sources (e.g., smoking habits, exposure to radiations, sun and nanoparticles, excessive alcohol intake, physical exercise, intake of drugs (e.g., contraceptive pills), mitochondrial and endothelial dysfunctions, iron overload, chronic inflammation, etc.) [3].

Obtaining evidence in vivo pathological OS still remains a great challenge [4,5]. Five classical axes of investigation using biological samples (whole blood, plasma, serum, urine, saliva, expired air) have been developed: measurement of both enzymatic (e.g., superoxide dismutase, glutathione peroxidases, etc.) and low molecular weight (vitamins C and E, glutathione, ubiquinone, thiol proteins, carotenoids, polyphenols, etc.) antioxidants, determination of trace elements (selenium, copper, and zinc), indirect ROS production evidenced by evaluating oxidative damage to lipids (lipid peroxides, isoprostanes, 4-hydroxynonenal), DNA (8-OH deoxyguanosine), and proteins (carbonyl group), or by identifying endogenous sources of ROS production (hyperglycemia, inflammation, iron overload, etc.) and, finally, use of a dietary questionnaire for estimating antioxidant intake [6]. In addition to being invasive (except the last one), all these approaches are time-consuming and require complex protocols, specific equipment (HPLC, ELISA, chemiluminescence, fluorescence and spectrophotometry, mass spectrometry, etc.). and qualified staff, thus making the majority of the analysis very expensive. Due to the great complexity of OS, none of them can claim to be a specific index of this biological process, except perhaps isoprostanes being considered as a “gold standard” of the lipid peroxidation process [7]. In order to bypass this problem, some authors have proposed the determination of the total antioxidant capacity (TAC) of biological samples such as whole blood and plasma. The most popular test is the ORAC (oxygen radical antioxidant capacity) assay which evaluates how antioxidants present in biological samples can inhibit, or not, physiological free radical species produced in a test tube. A very strong limitation of such an approach is that the plasma antioxidant capacity is dependent on uric acid and total protein concentrations of more than 70% [8,9].

A promising, but always under-explored way, to show OS is to use the skin as a matrix [10]. Having the largest surface area of the body, skin is the major organ target for oxidative stress, as it is continually exposed to external and internal “aggressions. As early as 1999, Kohen et al. [11] were the first to develop a noninvasive in vivo method for measuring at the skin level both low molecular weight antioxidants (LMWA) and lipid hydroperoxides levels as an indicator of oxidation status. For that, they used an electrochemical methodology (potentiometer) able to evaluate potential changes of reference and working electrodes immersed in an electro-conductive solution (FeCl_3_) applied to the skin. Later, Brainina’s group [12] determined both antioxidant (AOA) and oxidant (OA) skin activities using a K_3_[Fe(CN)_6_/]/K_4_[Fe(CN)_6_] mixture as a reduced/oxidized mediator (M) system, platinum as the working electrode, and electro-cardio cardiogram (ECG) electrodes as the reference. Ermakov et al. [13] developed resonance Raman spectroscopy (RRS) and reflection spectroscopy (RS) as optical methods applicable to the noninvasive detection of carotenoids in human skin. The authors showed that the skin carotenoid status can be a strong predictor of plasma carotenoid status and dietary intake of vegetables and fruits enriched in carotenoids [14,15]. Using electron spin resonance (ESR), a technology specifically dedicated to directly detect oxygenated free radicals, D’Errico et al. and Bourji et al. showed ROS production, respectively, on frog skin biopsies and fibrotic and non-fibrotic skin biopsies from patients with diffuse cutaneous systemic sclerosis [16,17]. Even if being specific for detection of free radical species, ESR technology has the great disadvantages to need biopsies, to be time consuming, being expensive, and requiring highly qualified staff.

Due to its easy method of application, we decided to develop an improved electrochemical method for evaluating SOSS by increasing sensibilities of both mediator M and microelectrodes. In addition to the examination of both method robustness and validation, we also investigated, for the first time, the possible existence of relationships between SOSS and blood concentration of classical OS biomarkers.

## 2. Materials and Methods

### 2.1. Reagents and Chemicals

Butylhydroxyanisol, 3,5-Di-*tert*-butylhydroxytoluene, sinapic, benzoic, salicylic, ferulic, and uric acids, catechol, cysteine, β-caroten, α-tocopherol, resveratrol, curcumin, glutathione, and bilirubin as antioxidants were all purchased from Sigma, Lezennes, France. Hydrogen peroxide, tert-butyl hydroperoxide, 2,2-diphenyl-1-picrylhydrazyl, 2,2′-zinobis(3-ethylbenzothiazoline-6-sulfonic acid, 2,2′-azobis(2-amidinopropane) dihydrochloride, potassium permanganate, and sodium hypochlorite as sources of oxidants production were also supplied by Sigma, Lezennes, France. Electrocardiogram (ECG) conductive gel was provided by Dermedics, Veauche, France.

The topical cream containing reduced coenzyme_10_ or CoQ_10_ (Sigma-Aldrich, Munich, Germany) as a bioactive antioxidant compound was prepared according to Kaci et al. [18]. Encapsulation of the antioxidant was performed thanks to nano–emulsions consisting of rapeseed oil (vegetable oil, Lesieur, Asnières-sur-Seine, France), rapeseed lecithin (Lecresoyaf F60 IP, Verdun, France) deionized water, and two polysaccharide polymers used as thickeners, xanthan gum (XG) from *Xanthomonas campestris* (Sigma-Aldrich, Munich, Germany), and carboxymethylcellulose sodium salt (CMC) (Alfa Aesar, Karlsruhe, Germany).

Food supplements fortified with vitamin C as synthetic (synthetic vitamin C supplement) or natural (Arkovital^®^ Pure’Energie containing natural vitamin C) composed of acerola berry (*Malpighia punicifolia* L. or *Malpighia glabra* L.), amla fruit (*Phyllanthus emblica* L.), goyave (*Psidium guajava* L.), holy basil leaf (*Ocimum tenuiflorum* L.), curry tree leaf (*Murraya koenigii* L.), and lemon (*Citrus limon* L) forms were supplied by Arkopharma, Carros, France. Each capsule of both forms contains 72 mg of vitamin C.

In Group III, vitamins C and E, reduced and oxidized glutathione, thiol proteins, selenium, copper, zinc, copper zinc/ratio, superoxide dismutase (SOD) and glutathione peroxidase (GPx), and lipid peroxides were assessed in plasma or whole blood according to analytical protocols as described earlier in detail by us [19,20,21]. Plasma isoprostanes were measured by Liquid chromatography-mass spectrometry (LC-MS) according to a homemade method [22]. Oxidative damage to DNA was evaluated by the measurement of urinary 8-hydroxydesoxyguanosine (8OH-dG) thanks to the kit developed by the Japan Institute for Control of Aging (Shizuoka, Japan). Total urinary polyphenols were measured according to Hoge et al. [6]. Both 8OH-dG and polyphenols were standardized to urinary creatinine. The participants also completed a self-administered food frequency questionnaire (FFQ) in order to estimate their dietary polyphenols intake [6].

### 2.2. Patients’s Consent

For each study presented in this paper, the trial objectives, study design, risks, and benefits were explained and written informed consent was obtained from all participants. As blood samples were needed for analysis of OS biomarkers, the full study protocol was approved by the institutional ethics committee of the Liège University Hospitals (reference 2017/342) and conducted in accordance with the 1964 Declaration of Helsinki and the European guidelines for good clinical practice.

### 2.3. Principle of SOSS Evaluation

**Figure 1:**
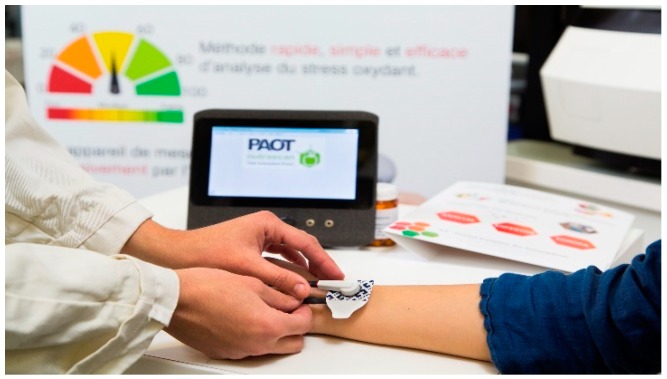
Photography of the electrochemical equipment (Oxystress Analyzer) allowing the measurement of SOSS in a non-invasive way. For details, see the Material and Methods section.

**Figure 2:**
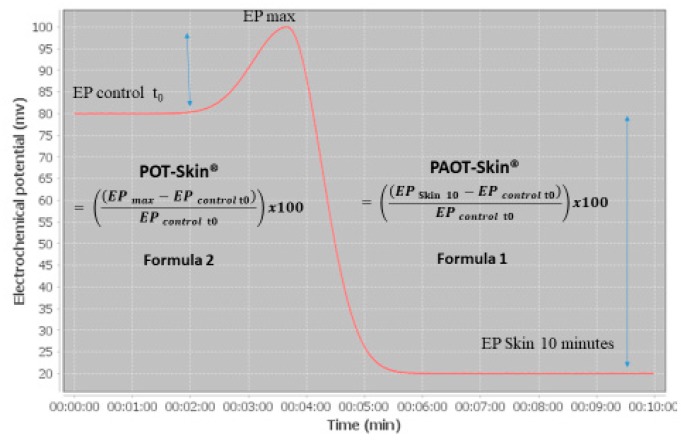
Kinetic curve of electrochemical potential changes during reaction of skin antioxidants and oxidants with oxidized/reduced iron forms (mediator M). PAOT-Skin Score^®^ was calculated as being the ratio PAOT-Skin^®^/POT-Skin^®^. EP control T_0_ = baseline potential of oxidized/reduced mediator M being at 80 nV.

SOSS (expressed as PAOT (Pouvoir AntiOxydant Total)-Skin Score^®^) reflecting the redox equilibrium between skin antioxidants and oxidants was measured using the equipment described in Figure 1 (Oxystress Analyzer). On subjects placed in a controlled temperature (20 °C) and humidity room (70%) during all experimentation, 12 cm^2^ skin areas, free of hair, were marked on the left forearm or back. Before analysis, the skin was cleaned with distilled water and dried with absorbent paper. The SOSS evaluation was performed by applying to the tested skin areas a patch constituting 1 mL ECG conductive gel containing both oxidized and reduced iron complex forms (mediator M) and being connected to both working and reference microelectrodes securely attached to the arms. Microelectrodes were recovered by four noble metal alloys, which is actually under patent FR1871986; 11.28.2018. Under these conditions, the initial potential of oxidized/reduced mediator M was around 80 nV, see Figure 2). SOSS was then estimated by registering for ten minutes the electrochemical potential shift in the gel mixture according to reactions between oxidized/reduced forms of mediator M with, respectively, skin antioxidants or AO (= PAOT-Skin^®^) and oxidants or O (= POT-Skin^®^).

M_ox_ + AO → M_red_ + AO_ox_

M_red_ + O → M_ox_ + O_red_

Results were calculated according to the two following formulas:

(1) $$\mathrm{PAOT}\;\left(\mathrm{Pouvoir}\;\mathrm{AntiOxydant}\;\mathrm{Total}\right)-{\mathrm{Skin}}^{®}=\;\left(\frac{\left({EP}_{10}\;-{EP}_{0}\right)}{{EP}_{0}}\right)\;\times \;100\%\;$$

(2) $$\mathrm{POT}\;\left(\mathrm{Pouvoir}\;\mathrm{Oxydant}\;\mathrm{Total}\right)-{\mathrm{Skin}}^{®}=\;\left(\frac{\left({EP}_{\max}-\;{EP}_{0}\right)}{{EP}_{0}}\right)\;\;\times \;100\%$$

where EP_0_ is the electrochemical potential at time 0, EP_max_ is the maximum electrochemical potential and EP_10_ is the electrochemical potential registered 10 min after the contact of the patch with the skin. Finally, the PAOT-Skin Score^®^ was calculated as the ratio PAOT-Skin^®^/POT-Skin^®^.

### 2.4. Determination of Antioxidant and Pro-Oxidant Activities Using Electrochemical Detection

For the determination of antioxidant and pro-oxidant activities, the same patch containing ECG conductive gel, mediator M, and microelectrodes as described in Section 2.3 was directly put in contact with pure antioxidants or oxidant system production in liquid form deposited on a tape strip. PAOT (Pouvoir AntiOxydant Total)-Score^®^ of antioxidants was calculated according to Equation (1), expressed as ascorbic acid equivalent (AAE)/L gel). When using oxidant sources, POT (Pouvoir Oxidant Total)-Score^®^ was calculated according to Equation (2), expressed as superoxide anion equivalent (SAE)/L gel × 1000.

### 2.5. Normal Reference PAOT-Skin Score^®^ Values and Stability Test

A first group (Group I) consisted of two hundred sixty-three healthily and fasted volunteers aged 20 to 65 years (116 females, 146 males, mean age and standard deviation (SD): 25.49 ± 6.22 years) was recruited for determining normal PAOT-Skin Score^®^ reference values. All subjects were not exposed to sun exposition before experimentation and did not apply products with an anti-wrinkle/anti-ageing/antioxidant action on the forearms during two weeks before the study started. They were also not allowed to consume fruits or fruit juices, or alcohol, or to practice sport during the 48 h preceding the experimentation. For intra-assay CV determination, we selected from this group nine volunteers (Group IIa) on whom the PAOT-Skin Score^®^ was evaluated on the same day 30, 60, 90, 120, and 150 min after the determination of the basal PAOT-Skin Score^®^ (T0). The same volunteers were also solicited to repeat during three straight weeks the measurement of the basal PAOT-Skin Score^®^ in order to determine the inter-assay CV (Group IIb).

### 2.6. Antioxidant Cream and PAOT-Skin Score^®^

**Figure 3:**
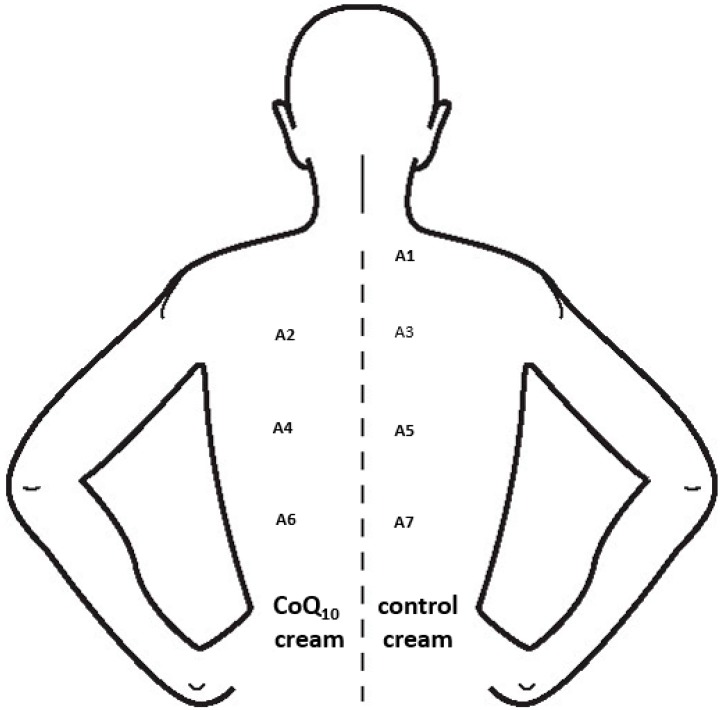
Localization of 12 cm^2^ areas of the back for the PAOT-Skin Score^®^ measurement after topical application of both control and reduced CoQ_10_-enriched creams. Zone A1: T0 corresponding to basal value; zones A2 and A3: measurement after 1 h; zones A4 and A5: measurement after 2 h; zones A6 and A7: measurement after 4 h.

A population of ten females (mean age: 22 years) was recruited for evaluating how the topical application of an antioxidant cream may affect the PAOT-Skin Score^®^ (Group III). All participants were asked not to take antioxidant supplementation for two weeks or to apply any products over their back on the evening before the experimentation. Both creams with or without reduced CoQ_10_ were topically applied on areas of 12 cm^2^ from the back according to the protocol described in Figure 3. In both conditions, PAOT-Skin Score^®^ (see the Material and Methods section) was evaluated 2, 4, and 6 h after cream application and compared to the measurement performed at T0.

### 2.7. Antioxidant Intake and PAOT-Skin Score^®^

A first intervention study (Group IV) was performed of nine healthy males (25–30 years) in order to check how the intake of orange juice could modulate the PAOT-Skin Score^®^. An amount of 400 mL of orange juice containing 264 mg vitamin C was ingested by each volunteer being fasted since the evening before the experimentation. The PAOT-Skin Score^®^ was analyzed before ingestion and 30, 60, 90, 120, and 150 min after orange juice intake.

In a second interventional study, six males (45 ± 7 years), fasted for 12 h, received 144 mg vitamin C (two capsules of 72 mg) either as synthetic (*n* = 3; synthetic vitamin C supplement, Group Va) or natural (*n* = 3; Arkovital^®^ Pure’Energie containing natural vitamin C, Group Vb) forms (Arkopharma, Carros, France). The PAOT-Skin Score^®^ was examined before and 1, 2, and 4 h after supplement intake.

### 2.8. Relationship between PAOT-Skin Score^®^ and Biomarkers of Oxidative Stress

Thirty subjects (12 females and 18 males, mean age: 42.9 ± 14.9 years) were selected to compare the PAOT-Skin Score^®^ with some demographic data and blood or urinary biochemical OS biomarkers (Group VI). Estimation of dietary vitamin C and total polyphenol intakes was achieved using a homemade food frequency questionnaire (FFQ) [6]. Dietary antioxidants were calculated by multiplying the consumption frequency of each item by the polyphenol content of selected portions. Data on the polyphenol content in foods were extracted from the Phenol-Explorer database which provides data on 502 polyphenol compounds in 452 foods.

### 2.9. Statistical Analysis

In the study performed in Group I, the distribution normality of PAOT-Skin Score^®^ was tested with the Shapiro–Wilk test. The comparison of all quantitative parameters was performed using the variance analysis (ANOVA) or the Kriskal–Wallis (KW) non-parametric test in the case of asymmetric distribution. In Groups III, IV, and V, *t* tests were determined using the GraphPad Prism 7. *p* values < 0.05 were considered as being significant. In Group VI, association between blood OS biomarkers and PAOT-Skin Scores^®^ was determined by the Pearson correlation coefficient or Spearman, in the case of asymmetric distribution. Results were considered as significant for *p* < 0.05.

## 3. Results

### 3.1. Electrochemical Detection of Antioxidant and Pro-Oxidant Activities.

Using the equipment described above, Table 1 shows that our electrochemical system was perfectly capable to evaluate antioxidant activities of pure molecules (1 mM) as measured by the electrode potential shift due to reactions between the oxidized mediator form (M_ox_) being in contact via the patch with antioxidants (PAOT-Score^®^). By contrast, the system was also able to detect pro-oxidant activities of systems producing, or not, physiological oxidants (0.1 mM) as measured by the electrode potential shift due to reactions between the mediator reduced form (M_red_) and oxidant molecules (POT-Score^®^).

### 3.2. Normal Reference Values (Group I)

**Figure 4:**
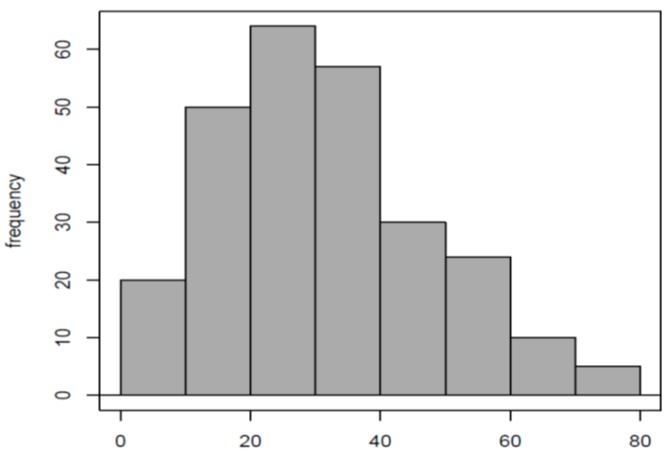
Normal distribution of the PAOT-Skin Score^®^ observed among 263 healthy volunteers (Group I). Reference values were calculated as ranging from 0–62.94.

Figure 4 shows that the distribution of PAOT-Skin Scores^®^ among the population of 263 healthy volunteers followed a typical Gaussian curve. This observation was confirmed by the result of the Shapiro–Wilk test (*p* = 0.1125). A mean value $\left(\bar{X}\right)$ of 31.09 and 16.25 as standard deviation (SD) were found. Due to normal distribution, we could apply the formula $\bar{X}\pm 1.96\times SD$ which allowed us to determine normal reference values for PAOT-Skin Score^®^ ranging from 0–62.94.

### 3.3. Intra- and Inter-Assays CV (Groups IIa et IIb)

Table 2 depicts that the basal PAOT-Skin Score^®^ (T0) was moderately affected if the measurement was repeated the same day 30, 60, 90, 120, and 150 min after the initial one. The intra-assay CV varied from 6.6–20.0% for individuals. Taking into account all subjects and all analysis times, a mean global intra-assay value of 12.46 ± 4.29% was found. As shown in Table 3, the inter-essay CV calculated on the basis of three measurements performed at weeks 1, 2, and 3 was 7.0 ± 2.5%.

### 3.4. Antioxidant Cream and PAOT-Skin Score^®^ (Group III)

**Figure 5:**
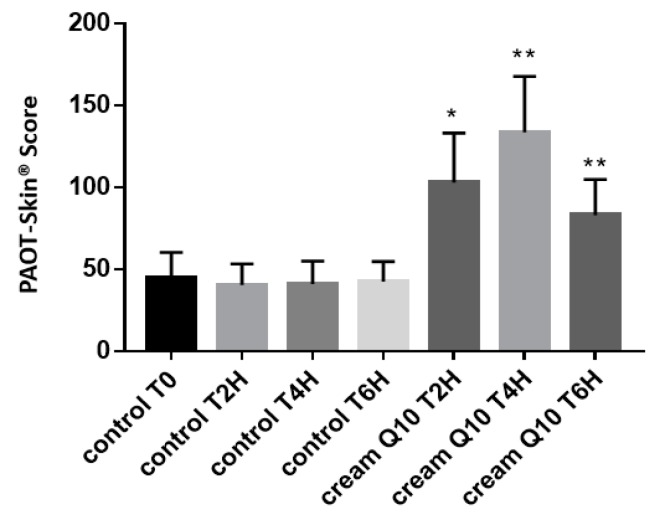
Effect of topical application of enriched (*n* = 12) or not-CoQ_10_ (*n* = 12) creams on the PAOT-Skin Score^®^ according to time (Group III). * *p* < 0.0001; ** *p* < 0.003.

Figure 5 depicts that the topical application of a cream containing reduced CoQ_10_ as an antioxidant resulted in a significant increase of the PAOT-Skin Score^®^. When compared to the basal value (T0), the skin antioxidant activity already increased by 95% 2 h after the cream application on the back to reach a maximal value of 150% at 4 h, then following by a drop to 56% after 6 h. By contrast, no modification of the skin antioxidant activity was detected when using the free antioxidant cream.

### 3.5. Food and Supplements Enriched in Vitamin C and PAOT-Skin Score^®^ (Group IV, Va, and VIb)

**Figure 6:**
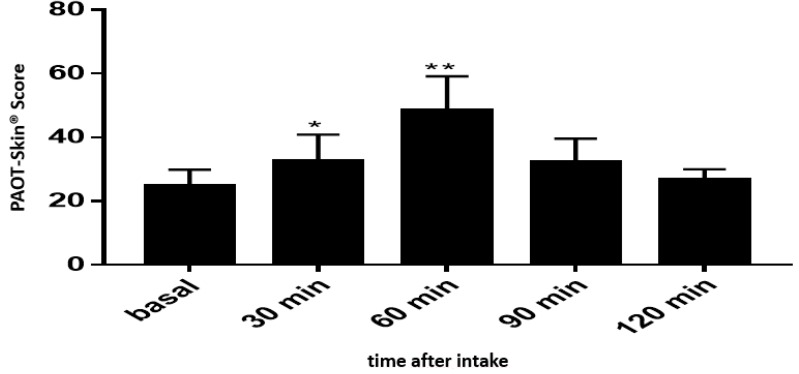
Intake of 400 mL orange juice containing 244 mg vitamin C and incidence on the PAOT-Skin Score^®^ according to time (Group V). * *p* = 0.047; ** *p* = 0.011.

Figure 6 shows that the basal PAOT-Skin Score^®^ significantly increased by 33.9% (*p* = 0.047) and 99.3% (*p* = 0.011), respectively, 30 and 60 min after the intake of orange juice (400 mL) containing 264 mg vitamin C (*n* = 9). After 90 and 120 min, the PAOT-Skin Score^®^ remained higher than the basal level, but in a non-statistical way.

**Figure 7:**
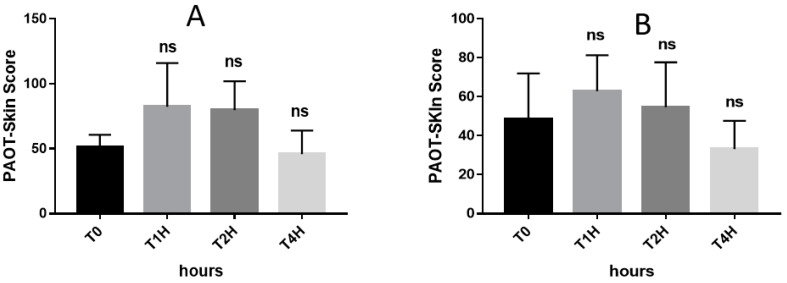
Effect of oral intake in natural vitamin C (Arkovital^®^ Pure’Energie containing Natural Vitamin C, *n* = 3, group VIb) (**A**) or synthetic (synthetic Vitamin C supplement, *n* = 3, group VIa) (**B**) at a dose of 144 mg on the PAOT-Skin^®^ Score according to time. Products supplied by Arkopharma, Carros, France.

As depicted in Figure 7A, the basal PAOT-Skin Score^®^ (51.05 ± 5.57) dropped to 82.33 ± 19.41 and 79.74 ± 12.84, respectively, 60 and 120 min after the intake of 144 mg natural vitamin C (*n* = 3) as a supplement. However, statistical analysis revealed that such increases were not significant. The PAOT-Skin Score^®^ returned to its basal value after four hours. Similar findings were found but to a lesser extent when using synthetic vitamin C as a supplement (Figure 7B).

### 3.6. PAOT-Skin Score^®^ and Oxidative Stress Biomarkers (Group VI)

Demographic data of volunteers (*n* = 30) are presented in Table 4. Men exhibited a significant higher age, weight, and body mass index (BMI) than women. Statistical analysis revealed that there was no correlation between the PAOT-Skin Score^®^ and all demographic data.

Table 5 describes the mean values ± SD observed for blood and urinary OS biomarkers. When compared to reference values defined as previously described by us [19,20,21], the concentration of all antioxidants was within the normal range. No significant difference was evidenced between men and women groups except for uric acid (*p* = 0.04). With respect to trace elements, copper and the Cu/Zn ratio were higher than the upper reference values when considering both men and women groups. Exceeding normal reference values was mainly due to the women, while men exhibited normal values. Of interest was to note that there was a statistical difference for these two parameters between men and women groups (*p* < 0.05). A similar finding was also evidenced for lipid peroxides as markers of increased oxidative damage to lipids. Finally, the mean value of the PAOT-Skin Score^®^ was higher than 17% in men when compared to women, but without reaching a statistical significance.

**Figure 8:**
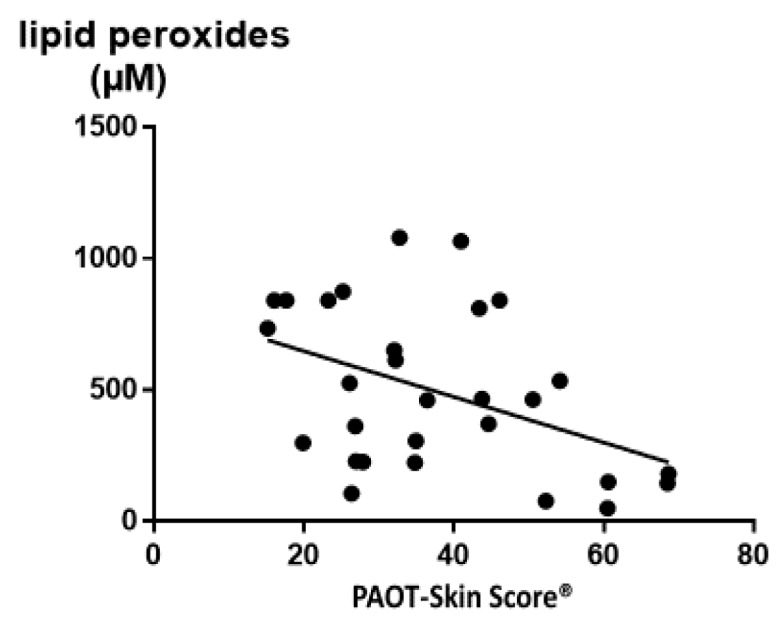
Negative correlation between the PAOT-Skin Score^®^ and blood lipid peroxides (r = −0.43, *p* = 0.020).

Statistical analysis revealed that blood OS biomarkers significantly associated to the PAOT-Skin Score^®^ were: γ-tocopherol/α-tocopherol ratio (r = 0.43, *p* = 0.020); copper (r = −0.42, *p* = 0.022); and the copper/zinc ratio (r = −0.49, *p* = 0.006) (Table 6). Figure 8 also evidenced the presence of a negative correlation between the PAOT-Skin Score^®^ and blood lipid peroxides. By contrast, no correlation was found with other blood OS biomarkers.

## 4. Discussion

Exploring skin as a matrix for evidencing in vivo OS may represent a promising and complementary analytical method to classical blood biomarker analysis. Major advantages for examining SOSS are multiple: easy access, large surface and, overall, the unique possibility to explore using electrochemical detection for the redox equilibrium between antioxidants and oxidants produced by continual skin exposure to both external and internal sources of ROS production.

Thanks to sensitive reference and working electrodes, our electrochemical method was perfectly able to register potential modifications induced by the interaction of pure antioxidants or sources of oxidant production, respectively, with the oxidized or reduced mediator M contained in the ECG gel (Table 1). Using ascorbic acid as a reference molecule, the PAOT-Score^®^ of antioxidants present in the skin, such as glutathione, uric acid, β-carotene, and α-tocopherol [24], were, respectively, 936, 329, 224, and 155 AAE/L gel). With respect to oxidant sources, it is of interest to note that sodium hypochlorite, tert-buytl hydroperoxide, and hydrogen peroxide, being naturally produced in the skin [11], exhibited the highest POT-Score^®^ when compared to superoxide anion taken as the reference system.

When applied to the skin, we confirmed that the ECG gel coupled to sensitive microelectrodes was able to evaluate the SOSS resulting from global potential modifications induced by interactions of oxidized/reduced M with both skin antioxidants (PAOT-Skin^®^) and pro-oxidants (POT-Skin^®^). The method robustness appeared to be adequate since respective CV inter- (54 measurements) and intra-assay (27 measurements) were of 12.46 ± 4.29% and 7 ± 2.5% (Table 2 and Table 3). This can be considered as acceptable for an in vivo methodology. Of high interest was the determination of normal PAOT-Skin Score^®^ reference values (0–62.94). To the best of our knowledge, such observations have never been conducted for skin OS status. Having in hand this parameter may, therefore, open the door to future clinical studies on populations known to be submitted to high OS, such as smokers or patients having type II diabetes, cardiovascular diseases, or cancer.

As antioxidants are one of the preferred way for reducing the ageing effect of skin, we investigated with our electrochemical system the impact of a cream enriched in reduced CoQ_10_ (ubiquinol) on the PAOT-Skin Score^®^. In addition to having potent antioxidant properties [25], the redox system of oxidized ubiquinone (ubiquinone)/reduced ubiquinone (ubiquinol) plays a major role in the mitochondrial electron transport chain. When compared to a control cream, topical application of reduced CoQ_10_ cream resulted, up to six hours after application, in a significant increase of the PAOT-Skin Score^®^ (150% at four hours) activity as shown in Figure 5. When compared to the technique described earlier by Ziosi et al. [26], for the routine evaluation of in vivo efficacy of antioxidant cream, our methodology is easier to use. Indeed, it did not require skin samples collected with adhesive tapes and, then, skin antioxidant extraction from the tapes for analysis by the luminescence method.

Among antioxidants, the roles of vitamin C in skin health have been reviewed in a recent paper by Pullar et al. [27]. In addition to an antioxidant effect, the presence of a high concentration of vitamin C in the skin has been associated with many potential functions, such as collagen formation, inhibition of melanogenesis, interaction with cell signaling pathways, and the modulation of epigenetic pathways. Figure 6 depicts that our electrochemical methodology was able to show the in vivo antioxidant effect of orange juice (264 mg vitamin C) intake as evidenced by the 33.9% (*p* = 0.047) and 99.3% (*p* = 0.011) increase of the PAOT-Skin Score^®^, respectively, 30 and 60 min after the 400 mL intake of the beverage. As shown in Figure 7, a similar increase could also be observed after oral intake of 144 mg natural or synthetic vitamin C in tablets. Statistical analysis revealed, however, that these increases were not statistically significant. This may be attributed to the small number of participants in our study. Similar experiments should be repeated in the long-term using higher doses in vitamin C. Costa et al. [28] reported an improvement of visible signs of skin ageing in men (*n* = 12) after an oral supplementation of 27 mg vitamin C but for six months. On the other hand, McArdel et al. [29] evidenced that oral vitamin C supplements, at 500 mg vitamin C/day, taken by 12 volunteers for eight weeks resulted in significant rises in plasma and skin vitamin C content.

The great novelty of the present study was to evaluate how the SOSS was associated, or not, with some epidemiological data and classical blood OS biomarkers. In Group VI, we were unable to highlight any association or correlation with all demographic data shown in Table 4. By contrast, results were more encouraging with OS blood biomarkers. Table 5 shows that all investigated parameters, including antioxidants, trace elements, and oxidative damage biomarkers, were in the normal reference values as established in our hospital routine laboratory. Exceptions were observed for plasma lipid peroxides and the Cu/Zn ratio with a level higher than the upper reference values. Of high interest was evidence of a negative and significant correlation between the PAOT-Skin Score^®^ and lipid peroxides (r = −0.43, *p* = 0.020) resulting from oxidative damage to lipids induced by ROS (Figure 8). We also found that the PAOT-Skin Score^®^ was negatively correlated with copper (r = −0.42, *p* = 0.022) and the Cu/Zn ratio (r = −0.49, *p* = 0.006), both parameters being considered as sources of increased oxidant production through the Fenton reaction (Table 6). All these observations are in agreement with other papers, with us showing that increased Cu/Zn ratio positively correlated with plasma lipid peroxides, more particularly in women taking contraceptive pills [30]. At least statistical analysis revealed a positive and significant correlation between the PAOT-Skin Score^®^ and the γ-tocopherol/α-tocopherol ratio (r = 0.43; *p* = 0.020). To our knowledge, no study has investigated in such a deep way potential relationships between SOSS and so large a battery of OS blood biomarkers. Using resonance Raman spectroscopy as the noninvasive methodology, one study has evidenced a significant and positive correlation (r = 0.62, *p* < 0.001) between total plasma carotenoid concentrations and skin carotenoid intensities [13]. However, the major drawback of this method was that it only detected a specific type of antioxidant while skin includes antioxidant enzymes and low-molecular weight antioxidants, such as vitamins C and E, ubiquinone, uric acid, and thiol compounds.

Even if the major limitation of our method was to give a global response, evidence of correlations between some OS blood markers and SOSS allowed, however, to consider our simple and noninvasive approach as a potential screening and inexpensive method for evidencing OS in pathologies such as cancer, diabetes, and neurodegenerative and cardiovascular diseases. Moreover, studies actually under investigation in our laboratory indicate that the PAOT-Skin Score^®^ could also be associated with the present electrochemical system adapted for liquid samples [31]. In Group III, data indicated a positive correlation between the PAOT-Urine Score^®^ and the polyphenols/creatinine ratio (r = 0.41; *p* = 0.017) as well as a negative correlation with the oxidized DNA/creatinine ratio (r = −0.48; *p* = 0.0092).

## 5. Conclusions

Our sensitive, but robust, electrochemical method may be considered as a useful tool for showing SOSS using the PAOT-Skin Score^®^, which reflects the redox balance between both skin antioxidant (AOA) and oxidant activities (OA). Its major advantages are to be noninvasive, non-expensive, and have very fast application. Of interest was the evidence of significant positive or negative correlations between the SOSS and some blood OS biomarkers (γ-tocopherol/(α-tocopherol, copper, copper/zinc, lipid peroxides). Integrating the present skin methodology among classical invasive blood biomarkers could be, therefore, useful to obtain a better comprehension of the OS role in human pathologies. In addition to such clinical application, our methodology offers many possibilities to test the impact of antioxidant creams, but also supplements or dietary products enriched in antioxidants on the modulation of SOSS.

## Figures and Tables

**Table 1:** PAOT-Score^®^ expressed as (AAE/L gel) × 1000) of pure antioxidants at a final concentration of 1 mM and POT-Score^®^ expressed as (SAE/L gel) × 1000) of six systems (0.1 mM) producing oxidants after 10 min of contact with ECG conductive gel containing the oxidized/reduced mediator M and connected to reference and working microelectrodes. PAOT-Score^®^ of ascorbic acid (AA) = 2699 ± 291; POT-Score^®^ of superoxide anion (SA) produced as described by Diaz-Uribe et al. [23] = 217 ± 20.

Antioxidant Samples	(AAE/L gel) × 1000
Resveratrol	1325 ± 87
Bilirubin	1070 ± 106
Curcumin	1011 ± 58
Catechol	942 ± 29
Glutathione	936 ± 60
Butylhydroxyanisol (BHA)	766 ± 31
Cysteine	660 ± 38
Enterodiol	488 ± 35
Sinapic acid	460 ± 30
Ferulic acid	456 ± 0.77
Benzoic acid	391 ± 2.97
Uric acid	329 ± 0.1
3,5-Di-*tert*-4-butylhydroxytoluene (BHT)	311 ± 5.68
Salicylic acid	278 ± 2.1
β-Carotene	224 ± 19
α-tocopherol	155 ± 1.72
Oxidant Sources	(SAE/L gel) × 1000
Sodium hypochlorite	10,691 ± 967
Tert-butyl hydroperoxide	7095 ± 433
Potassium permanganate (KMnO_4_)	2838 ± 225
Hydrogen peroxide (H_2_O_2_)	2529 ± 100
2,2′-Azinobis(3-ethylbenzothiazoline-6-sulfonic acid) (ABTS)	1483 ± 64
2,2’-Azobis(2-amidinopropane) dihydrochloride (AAPH)	1387 ± 18
2,2-Diphenyl-1-picrylhydrazyl (DPPH)	755 ± 43

**Table 2:** Intra-assay coefficient variability (intra-assay CV) of PAOT-Skin Score^®^ calculated (12.46 ± 4.29%) from a population of nine healthy volunteers (Group IIb).

	Basal Time	30 min	60 min	90 min	120 min	150 min	Mean	SD	CV%
Subject 1	12.56	16.8	16.08	14.37	14.73	12.14	14.45	1.69	11.7
Subject 2	18.45	13.86	10.44	13.46	15.69	14.76	14.44	2.42	16.7
Subject 3	39.61	34.26	37.91	35.87	34.37	31.54	35.59	2.62	7.3
Subject 4	14.55	13.84	12.99	12.61	11.9	12.67	13.09	0.87	6.6
Subject 5	17.71	19.75	19.14	18.79	16.46	13.89	17.62	1.98	11.2
Subject 6	13.53	13.31	13.21	13.06	12.9	13.07	13.18	0.2	15.3
Subject 7	65.84	53.74	52.7	40.02	38.69	40.97	48.66	9.75	20.0
Subject 8	17.71	19.75	19.14	18.79	16.46	13.89	17.62	1.98	11.2
Subject 9	28.24	29.85	29.73	28.2	25.5	20.23	26.96	3.33	12.3

**Table 3:** Intra-assay coefficient variability (intra-assay CV) of PAOT-Skin Score^®^ calculated (7.0 ± 2.5%) from a population of nine healthy volunteers (Group IIb).

	Week 1	Week 2	Week 3	Mean	SD	CV%
Subject 1	12.56	14.2	13.87	13.54	0.87	6.4
Subject 2	18.44	19.21	17.17	18.27	1.03	5.6
Subject 3	39.61	35.34	36.4	37.12	2.22	6.0
Subject 4	14.55	13.8	13.2	13.85	0.68	4.9
Subject 5	17.71	15.9	16.7	16.77	0.91	5.4
Subject 6	13.53	14.9	12.9	13.78	1.02	7.4
Subject 7	65.84	53.00	55.8	58.21	6.75	11.6
Subject 8	17.71	20.8	21.9	20.14	2.17	10.8
Subject 9	28.23	27.9	25.98	27.37	1.22	4.4

**Table 4:** Gender-specific demographic, biometric, medical, and dietary characteristics of the 30 volunteers participating to the study for evaluating relationship between PAOT-Skin Score^®^ and of OS biomarkers (Group VI).

	Men (*n* = 18)	Women (*n* = 12)	*p*-Value
Age (years)	46.22 ± 16.54	37.83 ± 10.36	0.089
Height	1.78 ± 0.05	1.67 ± 0.07	0.0005
Weight	81.61 ± 12.45	66.67 ± 12.10	0.046
BMI	26.28 ± 3.98	23.83 ± 3.78	0.38
Systolic blood pressure (mm Hg)	126 ± 21	116 ± 13	0.12
Diastolic blood pressure (mm Hg)	77 ± 9.49	79 ± 13.49	0.78
Smokers	-	-	-
Yes	0	0	-
No	18 (100)	12 (100)	-
Fruits intake (at least 2 servings/d)	-	-	-
Yes	4 (22)	4 (30)	0.50
No	14 (77)	8 (66)	-
Vegetables intake (at least 1 bowl/d)	-	-	-
Yes	4 (22)	5 (41)	0.25
No	14 (77)	7 (58)	-
Physical activity	-	-	-
Yes	7 (38)	7 (58)	0.29
No	11 (61)	5 (41)	-
Drugs intake	-	-	-
Yes	9 (50)	5 (41)	0.65
No	9 (50)	7 (58)	-
Antioxidant supplementation	-	-	-
Yes	4 (22)	3 (25)	0.86
No	14 (77)	9 (75)	-
Diet (and not diet) vitamin C intake (mg/d)	140 ± 61	135 ± 78	0.29
Diet (and not diet) polyphenols intake (mg/d)	2219 ± 908	2438 ± 2099	0.63
PAOT-Skin Score^®^	39.73 ± 15.78	33.85 ± 12.95	0.62

**Table 5:** Comparison of blood and urinary OS biomarkers, antioxidant intake and PAOT-Skin Score^®^ between males (*n* = 18) and females (*n* = 12) belonging to Group III.

	All Subjects (*n* = 30)	Men (*n* = 18)	Women (*n* = 12)	*p* Value	Reference Values
Vitamin C (µg/mL)	11.41 ± 3.34	11.84 ± 3.16	10.76 ± 3.62	0.44	6.2–18.8
Vitamin E (µg/mL)	10.77 ± 2.26	10.84 ± 2.42	10.66 ± 2.08	0.45	8.6–19.2
Total cholesterol (g/L)	1.73 ± 0.28	1.73 ± 0.25	1.72 ± 0.33	0.83	1.2–1.9
Vitamin E/cholesterol (µg/g)	6.09 ± 1.60	6.27 ± 1.8	6.33 ± 0.89	0.10	4.4–7
Gamma-tocopherol (µg/mL))	1.00 ± 0.36	1.04 ± 0.39	0.92 ± 0.30	0.43	0.28–2.42
β-carotene (µg/mL)	0.29 ± 0.19	0.25 ± 0.16	0.35 ± 0.22	0.33	0.05–0.68
Protein thiols (µM)	374.70 ± 31.34	377.17 ± 36.51	371.00 ± 22.46	0.74	310–523
Total glutathione (µM)	873.52 ± 132.17	876.89 ± 139.32	868,00 ± 125.94	0.80	717–1110
Oxidized glutathione (µM)	2.20 ± 2.05	2.52 ± 2.53	1.68 ± 0.67	0.39	1.17–5.32
GSHt/GSSG ratio	503.18 ± 176.39	480.83 ± 195.86	543.01 ± 134.69	0.78	111–747
SOD (IU/g Hb)	1842.40 ± 171.58	1838.39 ± 1561.10	1848.42 ± 199.66	0.50	785–1570
GPx (IU/g Hb)	51.80 ± 10.56	50.72 ± 9.84	53.42 ± 11.82	0.83	26–58
Uric acid (mg/dL)	5.34 ± 1.78	5.95 ± 1.82	4.41 ± 1.19	0.04	2.6–5.8
Selenium (µg/L)	80.47 ± 15.10	80.72 ± 16.36	80.10 ± 13.09	0.58	94–130
Copper (mg/L)	1.02 ± 0.34	0.90 ± 0.25	1.20 ± 0.40	0.12	0.8–1.20
Zinc (mg/L)	0.80 ± 0.13	0.82 ± 0.14	0.76 ± 0.11	0.18	0.7–1.20
Cu/Zn ratio	1.31 ± 0.52	1.09 ± 0.25	1.63 ± 0.66	0.05	1–1.17
Lipid peroxides (µM)	494.83 ± 309.45	369.61 ± 242.67	699.73 ± 305.95	0.05	0–432
Oxidized LDL (U/L)	50.90 ± 14.96	52.50 ± 17.06	48.50 ± 11.37	0.21	28–70
Antibodies against ox-LDL (IU/L)	389.57 ± 369.74	336.72 ± 317.92	468.93 ± 439.06	0.62	200–600
Isoprostanes (pg/mL)	186.41 ± 57.75	177.61 ± 52.62	200.82 ± 65.28	0.15	152–368
Oxidized DNA (µg/L)	11.15 ± 7.82	12.94 ± 8.45	8.23 ± 5.89	0.18	0–16
Creatinine (g/L)	1.64 ± 0.86	1.80 ± 0.86	1.40 ± 0.83	0.15	0.20–3.00
Oxidized DNA/creatinine (µg/g)	6.54 ± 2.13	6.97 ± 2.14	5.83 ± 2.02	0.10	0–20
Total urinary polyphenols (µg/L)	57429 ± 34130	62,478 ± 32,232.16	49,856 ± 39,895	0.63	ND
Total urinary polyphenols/creatinine (µg/g)	2297 ± 1413	36,451 ± 18,562	49,451 ± 39,057	0.20	ND
Daily intake vitamin C (mg/d)	138 ± 67	140 ± 62.48	135 ± 78.7	0.29	100
Daily intake polyphenols (mg/d)	2297 ± 1413	2219 ± 905	2438 ± 2099	0.64	1000
PAOT-Skin Score^®^	37.38 ± 15.06	39.73 ± 15.78	33.85 ± 12.95	0.62	0–62.94

**Table 6:** Significant correlations found between PAOT-Skin Score^®^ and blood OS biomarkers (Group III).

	PAOT-Skin Score^®^	*p*-Value
γ-Tocopherol/α-tocopherol	0.43	0.020
copper	−0.42	0.022
copper/zinc	−0.49	0.006
lipid peroxides	−0.43	0.020

## Abbreviations

AAE	Ascorbic acid equivalent
AOA	Antioxidant activity
CoQ_10_	Coenzyme Q_10_
CV	Coefficient variability
DNA	Deoxyribonucleic acid
ECG gel	Electrocardiogram gel
GPx	Glutathione peroxidase
M	Mediator
OA	Oxidant activity
PAOT	Pouvoir AntiOxydant Total
POT	Pouvoir Oxydant Total
OS	Oxidative stress
SOD	Superoxide dismutase
SOSS	Skin oxidative stress status
SOE	Superoxide anion equivalent
8-OH dG	8-Hydroxydesoxyguanosine
